# 25(OH)D levels in trained versus sedentary university students at 64° north

**DOI:** 10.1080/22423982.2017.1314414

**Published:** 2017-04-28

**Authors:** Scott P. Jerome, Kendra D. Sticka, Theresia M. Schnurr, Sally J. Mangum, Arleigh J. Reynolds, Kriya L. Dunlap

**Affiliations:** ^a^ Department of Chemistry and Biochemistry, University of Alaska Fairbanks, Fairbanks, AK, USA; ^b^ College of Health, University of Alaska Anchorage, Anchorage, AK, USA; ^c^ Novo Nordisk Research Foundation Center for Basic Metabolic Research, University of Copenhagen, Copenhagen, Denmark; ^d^ Department of Family Medicine, Pacific Northwest University of Health Sciences, Yakima, WA, USA; ^e^ Department of Veterinary Medicine, University of Alaska Fairbanks, Fairbanks, AK, USA; ^f^ Institute of Arctic Biology, University of Alaska Fairbanks, Fairbanks, AK, USA

**Keywords:** Cholecalciferol, exercise, endurance athletes, high latitude, vitamin D

## Abstract

**Purpose:** 25-hydroxyvitamin D (25[OH]D) deficiency is associated with compromised bone mineralisation, fatigue, suppressed immune function and unsatisfactory skeletal muscle recovery. We investigated the risk of 25(OH)D insufficiency or deficiency in endurance athletes compared to sedentary non-athletes living at 64° north.

**Methods:** University student-athletes (TS) and sedentary students (SS) volunteered to participate in this study. TS engaged in regular exercise while SS exercised no more than 20 minutes/week. Metabolic Equivalent of Task (MET) scores for participants were determined. Vitamin D intake was assessed using the National Cancer Institute’s 24-hour food recall (ASA24). Fasting plasma 25(OH)D levels were quantified via enzyme-linked immunosorbent assay.

**Results:** TS reported higher activity levels than SS as assessed with MET-minutes/week and ranking of physical activity levels (p < 0.05). The reported mean daily intake of vitamin D was higher in TS compared to SS (p < 0.05) while 25(OH)D plasma levels were lower in TS than in SS (p < 0.05). In total, 43.8% of the TS were either insufficient (31.3%) or deficient (12.5%) in 25(OH)D, while none of the SS were insufficient and 13.3% were deficient.

**Conclusion:** TS are at increased risk of 25(OH)D insufficiency or deficiency compared to their sedentary counterparts residing at the same latitude, despite higher vitamin D intake.

## Introduction

Vitamin D insufficiency and deficiency in adults and children has been described as endemic worldwide [1–5] and has been investigated extensively in the recent past. One of the earliest scientifically identified roles of vitamin D supplementation is the regulation of proper cellular calcium function in the prevention and treatment of rickets in children and osteomalacia in adults [6,7]. In addition to these roles in mineral balance and bone metabolism, vitamin D has pleiotropic effects in many human cells [8]. While consensus has not been reached, recent evidence suggests that vitamin D may play a significant role in overall health in addition to bone strength and density. Health issues linked to insufficient or deficient levels of vitamin D include diabetes [8,9], cancer [2], thyroid function [10], cardiac volume [11], immune system function [12], obesity [13], cardiovascular disease and metabolic syndrome [8]. Although further research is needed, researchers have demonstrated that vitamin D sufficiency is positively correlated with proper muscle function [4,14–17] and skeletal muscle recovery and regeneration [18], while deficiency is associated with muscle pain, weakness [1] and inadequate repair [18].

A rising number of studies suggest that many athletes do not maintain sufficient levels of 25-hydroxyvitamin D (25[OH]D) [19], although few studies have investigated this circumstance in relation to geographical latitude [20–22]. The prevailing hypothesis denotes that small sun angles in winter months at latitudes greater than 42° result in limited or insubstantial conversion of 7-dehydrocholesterol to vitamin D_3_ in the skin [1,23,24]. In a cross-sectional study of 2548 adults in Norway, Larose et al. described overall 25(OH)D deficiency (<50 nM) at 40%, with a seasonal shift of 64% in winter to 20% in summer [3]. Vitamin D levels in populations living at high latitudes are more likely to be inadequate as compared to populations living near the equator [25–27]. These findings, along with those suggesting that athletes are at greater risk of inadequacy than non-athletes, may prove to compound the overall risk of athletes living and training at high latitudes.

Our aim was to test the hypothesis that athletes living in Fairbanks, Alaska, at 64° north are at greater risk of vitamin D insufficiency and deficiency as compared to their sedentary counterparts living at the same geographic location.

## Methods

Approval for this study was secured by the Institutional Review Board of the University of Alaska Fairbanks (UAF, #492213-4) prior to data collection. Following an explanation of the study, including risks and benefits, we obtained written consent from all participants.

A power analysis using previous studies with athletes was completed prior to the recruitment of participants. Thirty-one male (n = 16) and female (n = 15) study participants, all non-pregnant and non-diabetic and 18–25 years of age, were recruited from the UAF student body. They consisted of two groups: trained student-athletes (TS; n = 16) and sedentary, non-athlete students (SS; n = 15). All TS participants were members of the UAF cross-country skiing and/or cross-country running teams. TS were engaged in regular endurance exercise for 10–20 hours per week for at least 3 months prior to data collection in preparation for National Collegiate Athletic Association competitions. SS were recruited through direct contact in entry-level science classes, fliers posted across the UAF campus and social media. A simple Google Forms questionnaire was used to pre-screen SS for current and past exercise habits. SS reported not engaging in regular moderate physical activity (defined as “physical activity that takes moderate effort and makes you breathe somewhat harder than normal”) for more than 20 minutes per bout, one bout per week, over the previous 3 months.

Participants completed a questionnaire that included health history, age, sex and race (Table 1). There were no significant differences between TS and SS with regards to age or sex. Study participants were predominantly white with no significant differences in race between groups.

**Table 1.** Demographic data for trained student athletes and sedentary, non-athlete students living at 64° north.

Parameter	Trained	Sedentary
n	16	15
Age^†^	20.1 ± 2.0	21.5 ± 2.1
*Gender*		
Male	8	8
Female	8	7
*Ethnicity*		
White	16	14
Black	0	0
Alaskan Native	0	3^‡^
Asian	0	0

Demographic data as reported on health history form.

^†^Data reported as group mean ± standard deviation.

^‡^Two participants of the sedentary group identified as both white and Alaskan Native ethnicity.

After removal of shoes, socks and heavy clothing, anthropometric measurements were taken by a registered nurse (Table 2). Using a TANITA TBF-300A body composition analyser (Tanita Corporation of America, Inc., Arlington Hills, IL) set to the standard position for all participants, weight and percentage body fat were measured.

**Table 2.** Anthropometric data for trained student athletes and sedentary, non-athlete students living at 64° north.

Measurement	Trained mean	Sedentary mean^†^	p-value
*Height (inches*)			
Male	70.48 ± 2.94	70.05 ± 2.65	0.7591
Female	65.42 ± 2.37	63.71 ± 3.85	0.3131
*Weight (lbs)*			
Male	154.6 ± 20.38	182.9 ± 31.66	0.0512
Female	131.7 ± 15.57	135.4 ± 41.11	0.8126
*Bodyfat (%)*			
Male	10.88 ± 4.02*	19.84 ± 6.15	0.0039
Female	23.45 ± 5.59	25.30 ± 11.06	0.6832
*Bodymassindex(kg/m^2^*)			
Male	21.84 ± 1.69*	26.04 ± 4.14	0.0188
Female	21.65 ± 2.04	23.44 ± 6.47	0.4686
*Basalmetabolicrate(kcal/day)*			
Male	1787 ± 153.4	1956 ± 218.0	0.0943
Female	1445 ± 77.27	1446 ± 190.7	0.9950

Data reported as group means ± standard deviation.

*Significantly different (p < 0.05).

^†^Female participants in this group, n = 7; all other groups, n = 8.

Both groups completed the International Physical Activity Questionnaire (IPAQ) Short Form in the presence of a researcher. The data were cleaned and analysed using IPAQ Guidelines for Data Processing and Analysis. Data from one TS and one SS participant were excluded based on this analysis. Metabolic equivalent of task (MET) scores in minutes per week were determined for each participant and classified into one of three physical activity categories: walking, moderate or vigorous.

Vitamin D intake was gleaned from dietary data collected using the National Cancer Institute’s 24-hour food recall (ASA24) system. Participants were instructed to record food and supplement (vitamin D) intake from one typical weekday and one typical weekend day. Data were analysed according to the ASA24 protocol.

Blood draws were performed during the last week of November and the first week of December. Participants were instructed to fast for 12 hours prior to the blood draw. Consumption of water was permitted during the fast. Blood samples were collected in EDTA tubes, placed on ice and centrifuged within 2 hours of the draw. Plasma was aliquoted and stored at −80**°**C for later analysis.

A commercially available enzyme-linked immunosorbent assay kit (Enzo Life Sciences, Farmingdale, NY) was used to measure 25(OH)D according to the manufacturer’s instructions. Briefly, 25(OH)D from plasma samples and alkaline phosphatase-conjugated 25(OH) vitamin D_3_ bind competitively to sheep monoclonal 25(OH)D antibodies. The antibodies then bind to donkey anti-sheep IgG coated on the interior surface of the plate wells. Excess material is washed out, a substrate is added causing remaining alkaline phosphatase conjugate to turn yellow and plate absorbance is read at 405 nm with an optical plate reader. Sample 25(OH)D concentrations were extrapolated from a standard curve.

Consensus has not been reached on what constitutes “optimal” levels of serum 25(OH)D, nor have definitions of sufficiency, insufficiency or deficiency been standardised. The Institute of Medicine classification for serum 25(OH)D concentrations was employed for this study: ≥50 nM = sufficient, 30 to <50 nM = insufficient and <30 nM = deficient. However, the Endocrine Society Guidelines state that a 25(OH)D level of 75 nM is required for sufficiency [28]. Öhlund et al. [29] cite several reports for their use of the following scale: ≥75 nM = optimal, 50 to <75 nM = suboptimal, 37 to <50 nM = insufficient and <37 nM = severely deficient. Although limited studies have reported optimal 25(OH)D levels for human athletic performance, it has been proposed that higher serum 25(OH)D levels (>100 nM) may improve athletic performance [30].

GraphPad Prism version 5.0d (GraphPad Software, Inc., La Jolla, CA) was used for data analysis. A two-tailed, unpaired t-test was applied in order to evaluate differences between groups with regards to anthropometric data, IPAQ categorical comparisons, overall caloric intake and plasma 25(OH)D concentration. A Mann–Whitney U test was used in order to assess physical activity levels and daily vitamin D intake for TS and SS. Male TS data were compared to male SS data. Female TS data were compared to female SS data. Differences were considered significant at p ≤ 0.05. Data are presented as mean ± standard deviation unless otherwise indicated.

## Results

### Subject characteristics

Table 1 outlines the demographics of the TS and SS participants. There were no significant differences between TS (n = 16) and SS (n = 15) in age (TS = 20.1 ± 2.0, SS = 21.5 ± 2.1) or sex (male TS = 8, female TS = 8, male SS=8, female SS = 7). All of the TS participants identified their ethnicity as white. Of the SS group, 14 indicated their race as white, one indicated Alaskan Native (AN) and two reported as both AN and white.

Analysis of anthropometric data (Table 2) revealed significantly lower percentage body fat and body mass index (BMI) in male TS (10.88 ± 4.02% and 21.84 ± 1.69 kg/m^2^) as compared to male SS (19.84 ± 6.15% and 26.04 ± 4.14 kg/m^2^). All members of the TS group had a BMI in the healthy range (18.6–25.0 kg/m^2^), while 10 of the SS group had a healthy BMI, three were classified as overweight (25.1–29.9 kg/m^2^) and two were classified as obese (≥30 kg/m^2^). Female TS did not show significant differences in percentage body fat or BMI compared to female SS. Furthermore, there were no significant differences in height, weight or basal metabolic rate between the TS and SS for either sex.

### Physical activity

**Figure 1.**
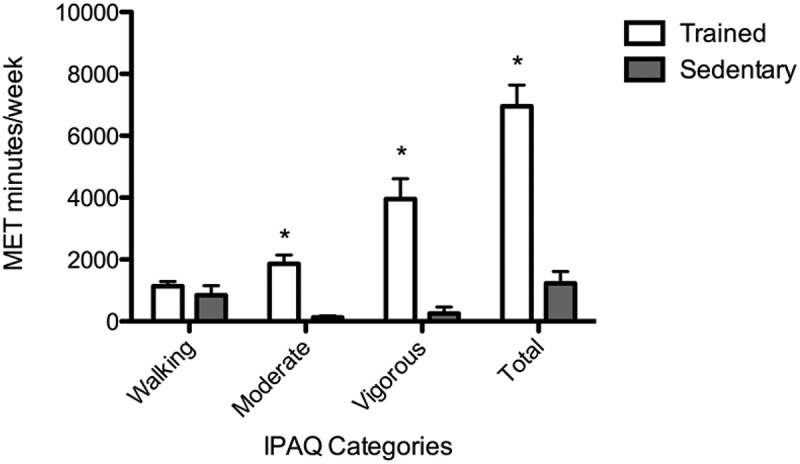
Physical activity (walking, moderate, vigorous and total) reported in metabolic equivalent of task-minutes/week for trained student athletes (open bars) and sedentary students (solid bars). Analysis was conducted according to International Physical Activity Questionnaire guidelines for data processing. Each bar represents the group mean ± standard error of the mean. *p < 0.05.

Walking, moderate, vigorous and total MET scores in minutes per week were determined for each study participant. IPAQ analysis revealed one TS participant with a moderate physical activity level while all other participants in the TS group sustained high physical activity levels. Conversely, SS participants demonstrated low physical activity levels, except for three with moderate and two with high levels. However, both MET-minutes/week (U = 7.00) and categorical ranking of physical activity levels (U = 17.5) were found to be significantly different between TS (n = 15) and SS (n = 14) groups. TS and SS groups demonstrated comparable walking MET scores (1134.10 ± 578.49 and 842.68 ± 1166.34; Figure 1). Moderate, vigorous and total METs were significantly greater in the TS group (1864.00 ± 1085.57, 3952.00 ± 2549.41 and 6910.10 ± 2669.78) compared to the SS group (127.14 ± 200.55, 257.14 ± 778.91 and 1226.96 ± 1450.22; Figure 1).

### Vitamin D intake

**Figure 2.**
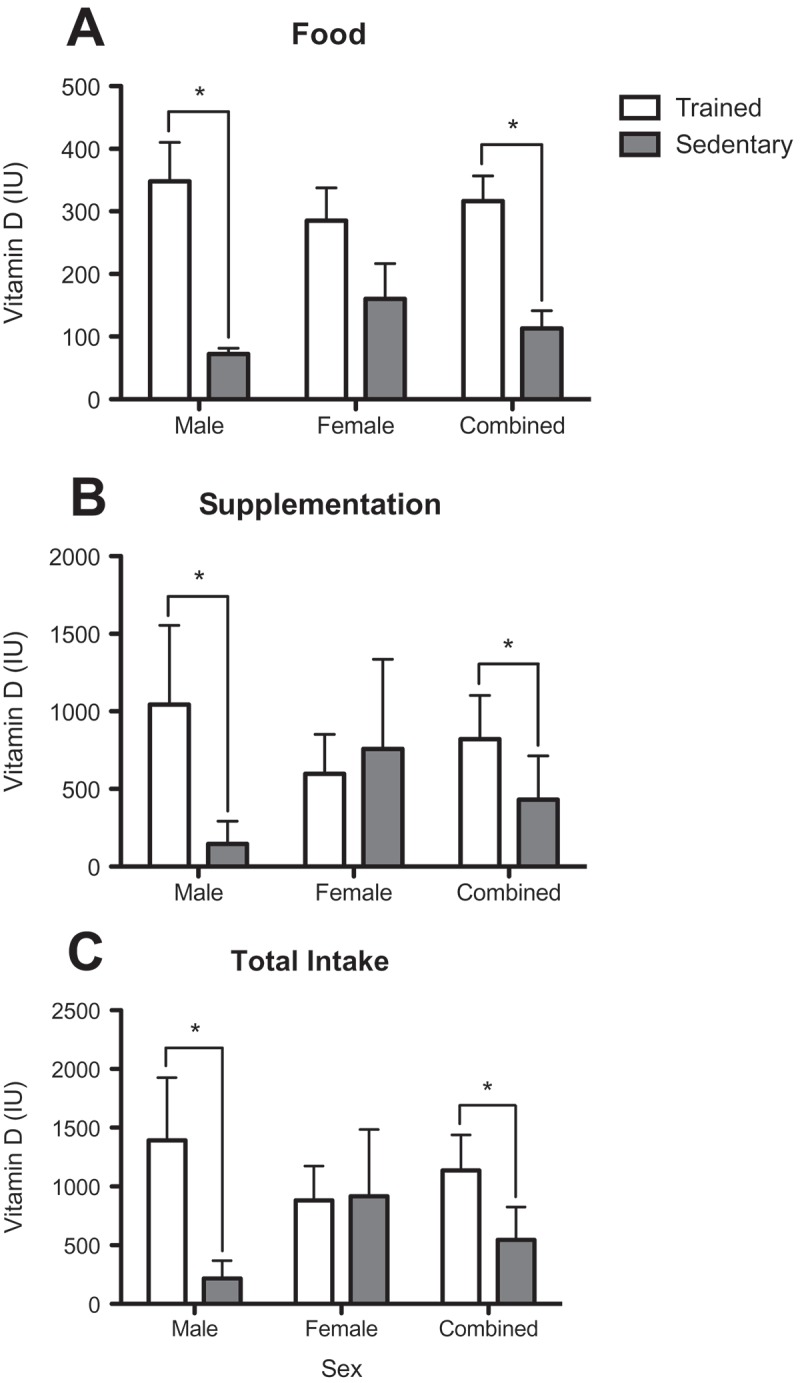
Vitamin D (IU) from (a) food, (b) supplementation and (c) total intake in trained student athletes (open bars) and sedentary students (solid bars). Data reported for males, females and both sexes combined. Data were obtained via the National Cancer Institute’s Automated Self-Administered 24-hour recall system. Each bar represents the group mean ± standard error of the mean. *p < 0.05.

Total vitamin D intake for each study participant was analysed as well as the source: food consumption or supplementation (Figure 2). Intake of vitamin D from food (Figure 2(a)) was significantly greater in the TS group (316.623 ± 160.79, U = 37) than the SS group (113.184 ± 110.13). Importantly, overall caloric intake was significantly higher for TS (3022 ± 951.04) as compared to SS (2041 ± 756.23). Male TS (348.00 ± 176.3, U = 8) consumed significantly more vitamin D from food compared to male SS (72.09 ± 26.88), whereas the intake of vitamin D from food in female TS and SS was not significantly different (285.2 ± 148.6 and 160.10 ± 150.40, U = 13). It is noteworthy that all participants had vitamin D intake from food sources below the recommended dietary allowance (RDA) of 600 IU per day.

A total of 14 out of 16 (88%) TS reported taking vitamin D supplements on one or both food recall days as compared to six out of 15 (40%) SS. Vitamin D supplementation (Figure 2(b)) was significantly greater for male TS (1044.00 ± 1445.00, U = 10.5) than male SS (146.60 ± 414.70) and for the sexes combined for TS (820.296 ± 1127.87, U = 67.5) than SS (431.533 ± 1091.28). There was no significant difference in vitamin D supplementation between TS and SS females (596.90 ± 723.6 and 757.10 ± 1532.00).

The total daily vitamin D intake (Figure 2(c)) was significantly greater for TS (1136.92 ± 1205.70, U = 50) than SS (546.52 ± 1089.93). Nine (56%) TS and three (20%) SS met or exceeded the RDA for this age group of 600 IU per day. The total daily IU intake for male TS (1391.72 ± 1512.33) was significantly greater than for male SS (222.09 ± 430.48), whereas there was no significant difference in total intake between female TS and SS (882.10 ± 824.30 and 917.3 ± 1502.00).

### Plasma 25(OH)D levels

**Figure 3.**
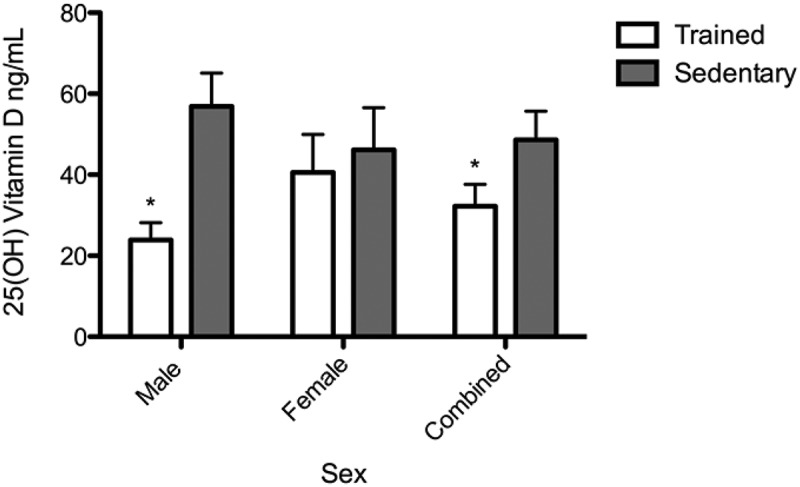
Fasting plasma 25-hydroxyvitamin D (25[OH]D) levels in trained student athletes (open bar) and sedentary students (solid bar). Data reported for males, females and both sexes combined. 25(OH)D levels were quantified by enzyme-linked immunosorbent assay. Each bar represents the group mean ± standard error of the mean. *p < 0.05.

Fasting plasma levels of 25(OH)D (Figure 3) were significantly lower in the TS group (32.24 ± 21.69) than in the SS group (51.92 ± 24.88). TS males (23.91 ± 11.97) demonstrated significantly lower levels of 25(OH)D as compared to SS males (56.93 ± 23.14), whereas TS and SS females had comparable concentrations (40.56 ± 26.57 and 46.20 ± 27.36). Furthermore, 44% of the TS participants were either insufficient (31%) or deficient (13%) in 25(OH)D, while none of the SS participants were insufficient and 13% were deficient. The remainder of the participants in both groups exhibited adequate levels.

## Discussion

The purpose of this study was to evaluate physical activity, vitamin D intake and fasting plasma 25(OH)D levels in TS compared to SS living in Fairbanks, Alaska, at 64° north. Sampling was conducted in November/December so that most, if not all, of the vitamin D in the study population would need to be obtained from diet. Low sun angles at this time of year in conjunction with the half-life of vitamin D would limit or eliminate conversion or storage of vitamin D from sunlight at 64° north. Physical activity was a lifestyle factor that was significantly different between the two groups. Most interestingly, TS consumed a greater total dietary intake of vitamin D, yet had significantly lower plasma levels of 25(OH)D as compared to SS. Our hypothesis that TS are at greater risk of 25(OH)D insufficiency or deficiency as compared to sedentary counterparts is supported by these data.

Ergocalciferol (vitamin D_2_) and vitamin D_3_ are fat-soluble secosteroid compounds that are essential to human health. Vitamin D_2_ is synthesised by plants and fungi and may serve as a dietary source for humans [10]. Vitamin D_3_ is produced by the conversion of 7-dehydrocholesterol in the skin via UV radiation. This transformation provides the most readily available source of vitamin D_3_ for human metabolism [29]. Vitamins D_2_ and D_3_ are inactive molecules that undergo two hydroxylation reactions for activation. In a step-wise fashion, vitamins D_2_ and D_3_ are converted to 25(OH)D in the liver before forming calcitriol (1,25[OH]_2_D) in the kidney. Concentrations of 1,25(OH)_2_D in serum are tightly regulated by parathyroid hormone, calcium and phosphate [31], with a circulating half-life of approximately 15 hours [32]. In contrast, 25(OH)D has a variable half-life of 15–45 days [33], making it a better indicator of vitamin D status and the standard measurement for research and clinical trials.

Vitamin D_3_ may also be ingested, although few unfortified foodstuffs provide sufficient concentrations to support adequate human health. Unfortified foods that are the exception include oily fish such as salmon, mackerel, herring and cod liver oil [23]. Oily fish are thought to have been an important source of vitamin D for indigenous peoples of the circumpolar north. In fact, shifts away from traditional subsistence diets towards more westernised diets are likely leading to the increasing prevalence of vitamin D deficiency and insufficiency in ANs [34]. Participants in this study were of predominantly European decent and did not live on a subsistence diet, nor did they consume higher than normal intakes of oily fish.

Vitamin D insufficiency and deficiency have been identified as worldwide health problems [23]. In a meta-analysis of 394 studies with over 33,000 subjects worldwide, Hagenau et al. [35] found mean 25(OH)D concentrations of 53.9 ± 1.0 nM, which is considered suboptimal or borderline insufficient. Furthermore, elite-level athletes appear to be at higher risk of vitamin D deficiency than others [14]. Explanations as to why this may be true include the utilisation of 1,25(OH)_2_D in muscle recovery [36], increased use of indoor training facilities [14] and increased use of sunblock and restrictive uniforms [28]. Moreover, it has been reported that exposure to winter sunlight at latitudes greater than 52° does not promote vitamin D_3_ synthesis in human skin from October through to March [24], and that those living at latitudes higher than 42° are at increased risk of seasonal insufficiencies or deficiencies in 25(OH)D [37].

Few studies, however, have investigated vitamin D status in outdoor athletes living at high latitudes. Most recently, a large study conducted over 4 years evaluated vitamin D status according to sun exposure and oral supplementation in Polish athletes training outdoors. As expected, athletes experienced the most severe hypovitaminosis in the winter months. Interestingly, increased winter sun exposure in athletes from northern climes – made possible by travelling to locations closer to the equator – improved vitamin D status more than oral supplementation [20]. Two studies that are more similar in scale and design to this one reported exacerbated hypovitaminosis in both soccer players training at 53° north [22] and rugby players training at 44° north [21] in the winter months. Hypovitaminosis was present, albeit to a lesser degree, in the summer months, indicating that the higher muscle masses of athletes require larger amounts of vitamin D [21]. Hence, athletes living and training in the subarctic are likely to be at even greater risk than the general population and athletes living and training at lower latitudes.

In this study, TS consumed a greater quantity of vitamin D via food sources than SS, which may be a result of food choices and/or significantly greater daily caloric intake (kcal) (3022.67 ± 951.04) compared to the SS group (2040.00 ± 756.32). Additionally, supplemental vitamin D intake was greater in TS than SS in both numbers of individuals taking vitamin D and the amount taken. Despite the higher intake, TS demonstrated significantly lower levels of plasma 25(OH)D compared to SS. Furthermore, nearly half of the TS participants had insufficient or deficient levels of 25(OH)D, while none of the SS participants were insufficient, and only two were deficient.

The data demonstrate that male TS have significantly lower 25(OH)D levels than male SS; females showed no difference between the groups. We speculate that this discrepancy between the sexes was a result of significant differences in body composition seen in the males but not the females. Research on body composition and vitamin D status indicates that both adiposity and reduced mean muscle mass contribute to vitamin D deficiency. For instance, obesity has long been associated with low 25(OH)D status [6], despite previous animal studies that established adipose tissue as the major storage site for vitamin D_3_ [38]. Epidemiological studies have demonstrated lower vitamin D levels being associated with higher BMI [39,40]. Our data are in contrast to this; however, any conclusion based on body fat would be difficult in the current study, given that only five of the study subjects were classified as overweight or obese. Research by Ko et al. found that men with a lower appendicular skeletal muscle mass index (ASMMI) score were more likely to be vitamin D deficient [41]. The authors conclude that a positive relationship between 25(OH)D and muscle mass in men may exist. Our results are also in conflict with these findings because we would expect our TS males to have higher muscle masses than their sedentary counterparts. The BMI of well-trained athletes is influenced primarily by their body fat content, as they tend to display optimal muscle mass [42]. However, it is important to note that ASMMI and percentage body fat are not identical measurements. A larger sample size would provide clarity regarding the relationship between hypovitaminosis, muscle mass and body fat composition.

Notwithstanding the small sample size, we showed significantly lower vitamin D levels in trained athletes living at high latitudes compared to their sedentary counterparts, despite higher vitamin D intake. Our data suggest that this risk is more pronounced in males than in females. This study contributes to the small but growing body of evidence that athletes living at high latitudes are at an even greater risk of vitamin D insufficiency or deficiency than other individuals living at the same latitude. Further investigations are needed in order to determine whether lower levels of plasma 25(OH)D in TS are due to an enhanced rate of skeletal muscle repair and/or other mechanisms that are unique to endurance athletics. These findings suggest that athletes living at high latitudes would benefit from regular vitamin D screening and oral supplementation.
